# Supplementary material for: Structural insight on the mechanism of an electron-bifurcating [FeFe] hydrogenase
Source: eLife. 2022 Aug 26;11:e79361. doi: 10.7554/eLife.79361 (PMC9499530; doi:10.7554/eLife.79361)
Supplement: Supplementary file 1. — (a) Table summarizing the buried surface area (in Å2), number of salt bridges and number of hydrogen (H-)bonds between the various interacting partners in the heterododecameric Hyd(ABC)4 complex. (b) Table summarizing the cryo-EM data collection and refinement statistics of the four structural models presented in this work: (1) the D2 tetramer of trimers model (PDB ID: 7P5H), (2) the bridge closed forward model (PDB ID: 7P8N), (3) the bridge closed reverse model (PDB ID: 7P91), and (4) the open bridge model (PDB ID: 7P92). [file elife-79361-supp1.docx]

Supplementary File 1 for:

**Structural insights on the mechanism of the electron-bifurcating [FeFe] hydrogenase from *Thermotoga maritima***

Chris Furlan^1†^, Nipa Chongdar^2†§^, Pooja Gupta^1^, Wolfgang Lubitz^2^, Hideaki Ogata^3,4^, James N. Blaza^1*^, James A. Birrell^2*^

Correspondence to: jamie.blaza@york.ac.uk, james.birrell@cec.mpg.de

**Contents**

Supplementary file 1a and 1b

**Supplementary file 1a. Interactions between subunits** **in *Tm*HydABC.**

| Interacting partners | Buried surface area (Å^2^) | Salt bridges | H-bonds |
| --- | --- | --- | --- |
| HydA-HydB | 1,232 | 6 | 4 |
| HydA-HydC | 608 | 1 | 1 |
| HydB-HydC | 1,403 | 2 | 5 |
| HydB-HydA’ | 407 | 0 | 2 |
| HydA-HydA’ | 2,280 | 14 | 8 |
| HydA-HydA’’ | 780 | 10 | 4 |

**Supplementary file 1b. Cryo-EM data collection and refinement statistics.**

|  | D2 tetramer | Bridge closed forward | Bridge closed reverse | Open bridge |
| --- | --- | --- | --- | --- |
| PDB-ID | 7P5H | 7P8N | 7P91 | 7P92 |
| EMD | 13199 | 13254 | 13257 | 13258 |
| Magnification | 165’000 | | | |
| Voltage (kV) | 300 | | | |
| Electron exposure (e–/Å^2^) | 57 | | | |
| Defocus range (μm) | -1 to -3 in 0.35 increments | | | |
| Pixel size (Å) | 0.824 | | | |
| Symmetry imposed | D2 | C1 | C1 | C1 |
| Initial particle images (no.) | 885k | 558k | 558k | 558k |
| Final particle images (no.) | 279k | 109k | 109k | 245k |
| Map resolution (Å) | 2.3 | 2.8 | 2.8 | 2.7 |
| FSC threshold | 0.143 | | | |
| Map sharpening *B* factor (Å^2^) | -80 | -78 | -78 | -86 |
| Initial model used | Complex I homology model (*4*) | | | |
| Nonhydrogen atoms | 40468 | 21429 | 21429 | 10545 |
| Protein residues | 5060 | 2681 | 2681 | 1322 |
| Ligands | 40 | 23 | 23 | 12 |
| Bond lengths (Å) | 0.005 | 0.004 | 0.004 | 0.004 |
| Bond angles (°) | 0.632 | 0.526 | 0.556 | 0.535 |
| Ramachandran outliers (%) | 0.00 | 0.08 | 0.04 | 0.00 |
| MolProbity score | 1.76 | 1.48 | 1.61 | 1.36 |
| Clashscore | 4.70 | 4.38 | 5.92 | 4.76 |
| Poor rotamers (%) | 2.33 | 0.00 | 0.00 | 0.09 |
| CaBLAM outliers (%) | 1.84 | 2.08 | 2.11 | 2.07 |
